# ALK Kinase Domain Mutations in Primary Anaplastic Large Cell Lymphoma: Consequences on NPM-ALK Activity and Sensitivity to Tyrosine Kinase Inhibitors

**DOI:** 10.1371/journal.pone.0121378

**Published:** 2015-04-13

**Authors:** Federica Lovisa, Giorgio Cozza, Andrea Cristiani, Alberto Cuzzolin, Alessandro Albiero, Lara Mussolin, Marta Pillon, Stefano Moro, Giuseppe Basso, Angelo Rosolen, Paolo Bonvini

**Affiliations:** 1 Clinica di Oncoematologia Pediatrica di Padova, Azienda Ospedaliera-Università di Padova, Padua, Italy; 2 Dipartimento di Scienze Biomediche, Università di Padova, Padua, Italy; 3 Dipartimento di Scienze del Farmaco, Università di Padova, Padua, Italy; 4 CRIBI, Università di Padova, Padua, Italy; 5 Istituto di Ricerca Pediatrica Città della Speranza, Padua, Italy; Istituto dei tumori Fondazione Pascale, ITALY

## Abstract

ALK inhibitor crizotinib has shown potent antitumor activity in children with refractory Anaplastic Large Cell Lymphoma (ALCL) and the opportunity to include ALK inhibitors in first-line therapies is oncoming. However, recent studies suggest that crizotinib-resistance mutations may emerge in ALCL patients. In the present study, we analyzed ALK kinase domain mutational status of 36 paediatric ALCL patients at diagnosis to identify point mutations and gene aberrations that could impact on NPM-ALK gene expression, activity and sensitivity to small-molecule inhibitors. Amplicon ultra-deep sequencing of ALK kinase domain detected 2 single point mutations, R335Q and R291Q, in 2 cases, 2 common deletions of exon 23 and 25 in all the patients, and 7 splicing-related INDELs in a variable number of them. The functional impact of missense mutations and INDELs was evaluated. Point mutations were shown to affect protein kinase activity, signalling output and drug sensitivity. INDELs, instead, generated kinase-dead variants with dominant negative effect on NPM-ALK kinase, in virtue of their capacity of forming non-functional heterocomplexes. Consistently, when co-expressed, INDELs increased crizotinib inhibitory activity on NPM-ALK signal processing, as demonstrated by the significant reduction of STAT3 phosphorylation. Functional changes in ALK kinase activity induced by both point mutations and structural rearrangements were resolved by molecular modelling and dynamic simulation analysis, providing novel insights into ALK kinase domain folding and regulation. Therefore, these data suggest that NPM-ALK pre-therapeutic mutations may be found at low frequency in ALCL patients. These mutations occur randomly within the ALK kinase domain and affect protein activity, while preserving responsiveness to crizotinib.

## Introduction

Anaplastic Large Cell Lymphoma (ALCL) represents a distinct subset of T-cell non-Hodgkin lymphoma (NHL), accounting for about 10–15% of childhood lymphomas [1]. The relative rarity of this tumour has limited the number of large prospective clinical trials for treatment optimization, and current therapeutic strategies are still based on the use of combined intensive chemotherapy. Despite current treatments achieve an event-free survival around 75%, the outcome of relapsed patients is less than 60% [2] and more effective therapeutic strategies are demanding.

Anaplastic Lymphoma Kinase (ALK) is a receptor tyrosine kinase that was originally described in t(2;5)(p23;q35)-positive ALCL as part of the NPM-ALK fusion protein [3]. Although the physiological function and regulation of full-length ALK receptor is still poorly characterized, aberrant expression of constitutively activated NPM-ALK has been clearly established as the leading cause of ALK-positive ALCL [4]. Tumours bearing *ALK* gene translocations, amplification or activating point mutations, other than ALCL, have been also identified, including non-small cell lung cancer (NSCLC) [5], Inflammatory Myofibroblastic Tumour (IMT) [6] and neuroblastoma [7]. Compelling studies have indicated that all these malignancies are partially or fully dependent on ALK kinase activity for proliferation and survival [7,8,9], as inhibition of ALK or downregulation of its expression yields potent anti-tumour efficacy both *in vitro* and *in vivo* [10].

In this context, the ALK kinase inhibitor crizotinib has been approved for the treatment of ALK-rearranged malignancies, and is now considered the standard of care for both early- and advanced-stage NSCLC patients [11,12]. More recently, crizotinib has entered Phase I/II clinical trial for the treatment of young patients with relapsed or refractory solid tumors and ALCL (ClinicalTrials.gov, NCT00939770, Children’s Oncology Group, United States), given to the favourable toxicity profiles and objective response rate demonstrated [13]. For these reasons, the opportunity to use crizotinib as part of first-line therapy in children with ALCL is presently being considered, although failure after treatment, like that reported in a small number of NSCLC and IMT patients [11,12,14], or described by other previous clinical experiences [15,16,17], cannot be overlooked. With kinases, in fact, relapse may be linked to drug-resistance mutations in the catalytic domain, both when acquired *de novo* and resulting from selection of pre-existing subdominant clones [18,19,20]. The knowledge gained about drug resistance in cancer has shown that minor mutated cell populations can be identified in patients before the onset of treatment, including those that simply promote tumour progression or contribute to resistance [21]. Secondary mutations associated with resistance via reduced inhibitor binding [22,23,24] or increased kinase activity have been described in ALK-positive ALCL as well [25,26,27]. However, their presence at diagnosis has never been investigated, likewise their evolution and impact.

In the present study we performed mutational analysis of NPM-ALK kinase domain in paediatric ALCL tumours, to identify point mutations and gene aberrations that could result in changes of NPM-ALK expression and oncogenic activity. Detection of variants was performed by ultra-deep sequencing, in order to assess, at the time of diagnosis, the presence of subclonal mutations not distinguished by conventional Sanger sequencing.

The results of this study demonstrated that aberrations of *NPM-ALK* gene, although uncommon in naïve patients, included both missense and INDEL mutations, which generated low-active and inactive fusion proteins. Functional validation of selected mutants was performed by expressing recombinant proteins in the presence or absence of active NPM-ALK kinase, coupled to structure-based computational analysis of ALK catalytic domain. Biochemical results and molecular modelling data confirmed the predicted silent nature of INDELs, and revealed new insights on ALK conformational changes upon single amino acid substitution. Nevertheless, we also found that INDEL mutations present at the time of diagnosis affected constitutive NPM-ALK kinase activity *in vitro*, by forming nonfunctional heterocomplexes and increasing the sensitivity to specific inhibition.

## Materials and Methods

### Patients, samples and cell lines

A total of 36 tissue samples from ALK-positive ALCL patients, enrolled between December 2000 and September 2010 in AIEOP-LNH-97 or ALCL-99 treatment protocols, were included in this retrospective analysis. The study was approved by the ethic committee of Azienda Ospedaliera di Padova. In compliance with the Helsinki Declaration, informed written consent was obtained from parents or legal guardians on behalf of the children enrolled in the study [28]. Diagnosis was centrally reviewed by the AIEOP pathologists and further characterized by means of RT-PCR for t(2;5)(p23;q35) translocation [29]. Median age at diagnosis was 9.1 years (range between 3.6 months to 17.5 years), 23 cases were males and 13 females. Most of the cases represented common type ALCL (42%) and, based on St Jude classification, 92% were stage III-IV [30]. For the functional studies, COS7 and HEK-239T cells were grown in RPMI 1640 and DMEM medium, respectively, supplemented with 10% FCS, 2 mM glutamine (Gibco, Life Technologies Co., Carlsbad, CA, USA), 100U/ml penicillin and 100 μg/ml streptomycin (SIGMA-Aldrich Co., St. Louis, MO, USA).

### Reagents and antibodies

PF-02341066 (Crizotinib) and NVP-TAE684 (TAE684) were purchased from Selleckchem (Selleck Chemicals, Houston, TX, USA), dissolved in DMSO and stored at -20°C. The antibodies used for Western blot analysis were specific for STAT3^Y705^, ALK^Y1604^, ALK^Y1278/Y1282/Y1283^, c-*myc* epitope (rabbit) (used at 1:1000 dilution, Cell Signaling Technology, Inc., Danvers, MA, USA); STAT3 (1:1000, Santa Cruz Biotechnology, Inc., Santa Cruz, CA, USA); ALK, V5 epitope, c-*myc* epitope (mouse) (1:2000, Invitrogen, Life Technologies Co); γ-tubulin (1:5000, SIGMA-Aldrich). DAPI nucleic acid stain, and fluorophore-conjugated goat anti-rabbit Alexa488 and goat anti-mouse Alexa546 antibodies were bought from Molecular Probes (1:500, Molecular Probes, Life Technologies Co.). Horseradish peroxidase-conjugated sheep anti-mouse or donkey anti-rabbit antibodies, used at 1:2000 dilution, were purchased from GE Healthcare (GE Healthcare Life Sciences, Uppsala, Sweden). Protein G-sepharose Fast-Flow beads were from GE Healthcare as well. For Western blot analysis, proteins were quantified by BCA protein assay (Pierce Chemical, Co., Rockford, Illinois, USA), transferred to nitrocellulose membranes (Whatman, GE Healthcare Life Sciences) and visualized by using PerkinElmer chemiluminescence reagents (PerkinElmer Inc., Waltham, MA, USA), Amersham Hyper-film ECL (GE Healthcare Life Sciences) and Carestream Kodak Autoradiography chemicals (Sigma-Aldrich).

### RT-PCR and amplicon library preparation

Total RNA was isolated using TRIzol reagent (Invitrogen) and RT-PCR was performed as reported previously [29]. ALK kinase domain coding region, corresponding to exons 22–25, was amplified using fusion primers, consisting in a target-specific sequence on the 3’-end, an adapter sequence on the 5’-end and a different MID sequence for each primer pair, according to manufacturer’s guidelines (S1 Table and S2 Table). Negative and positive controls for mutated *ALK* gene used were commercially available human ALCL (KARPAS-299) and neuroblastoma (SH-SY5Y) cell lines, respectively. Amplicon products were quantified using Quantity One software (Bio-Rad Laboratories Inc., Hercules, CA, USA) and pooled at an equimolar ratio. Each sample was run on agarose gel, purified by QIAquick gel extraction kit (Qiagen Co., Hilden, Germany) and diluted to a final concentration of 10^7^ PCR fragment molecules/μl.

### Next-generation sequencing

Amplicon ultra-deep sequencing was performed using Roche 454 Genome Sequencers GS FLX and GS Junior (Roche Applied Science, Penzberg, Germany). The amplicon-PCR-derived fragments were annealed to carrier beads and clonally amplified by emulsion PCR (emPCR), according to the manufacturer’s protocol. The beads carrying single-stranded DNA templates were enriched, counted and deposited into the PicoTiterPlate for sequencing.

454 sequence data have been deposited in the European Nucleotide Archive (ENA, http://www.ebi.ac.uk/ena/data/view/) under the accession numbers ERS622534 and ERS622535.

### Data analysis and detection of variants

All data were generated using the GS Sequencer software version 2.5.3 (Roche Applied Science), and amplicon pipeline analysis was performed using default settings of the GS RunBrowser software version 2.5.3 (Roche Applied Science). Sequence alignments and variant detection was performed using GS Amplicon Variant Analyzer (AVA) 2.7 (Roche Applied Science), in combination with a blast-based pipeline for low frequent large INDELs detection (CRIBI Genomics, University of Padova, Padova, Italy). AVA software filters were set to display sequence variances represented even by a single read, using human NPM-ALK kinase mRNA sequence (GenBank U04946.1) for reference. Point mutations (single nucleotide polymorphisms, SNPs) were accepted when present with a frequency of at least 0.5% in both forward and reverse reads, whereas INDELs were considered when validated by both software, regardless of frequency. INDEL consensus sequences were analyzed using the mRNA-to-genomic alignment program Spidey (http://www.ncbi.nlm.nih.gov/spidey) and manually reviewed.

### Molecular modelling and dynamics simulation

ALK mutants were analyzed through the MOE Protein Align tool with BLOSUM 62 as substitution matrix. The homology models were obtained through the MOE homology modelling tool, using human wild-type (WT) and mutants R1275Q and F1174L ALK crystallographic structures (PDB code 3LCT, 4FNX and 4FNW, respectively) as homologous templates [31]. The models have been generated using AMBER99 forcefield, in the presence of ADP docked to the template active site, while water molecules and other cofactors have been removed.

The protonation state of ALK R308-Ins8 and R308-Ins12 tyrosine kinase models were evaluated with Protonate3D (T = 300K, pH = 7) within MOE and Protonate within AmberTools 1.5.

The missing residues of WT and R1275Q structures were built using the same approach described above. We used tLeap and Amber FF99SB to parameterized the 'Ins' protein models and solvated them in TIP3P water boxes, adding counterions (Na^+^; Cl^-^), whereas the point mutant models were parameterized with CHARMM27 forcefield. ClickMD has been used as molecular dynamic platform for NAMD 2.9 minimization (100000 step, conjugated-gradient method), equilibration (0.5 ns, alpha carbon positional restrains) and production phase (100 ns NVT, P = 1atm, T = 300K) of the molecular systems through 100000 conjugated gradients method. ACEMD v2728 has been used as molecular dynamics engine on nVidia GeForce GTX680 computational platform. Finally, the analysis of the resulting trajectories was based on RMSD overtime, RainbowRMSD, heatmaps and distance analysis employing VMD 1.9.1, RMSD Trajectory Tools 2.01, RAINBOWRMSD and NRGPLOT.

### Generation of mutant constructs

The pcDNA3 plasmid containing WT NPM-ALK was obtained from the original pSRα-tkneo-NPM-ALK plasmid (a kind gift from Dr. S. Morris, S. Jude Research Hospital, Memphis, TN, USA), whereas all the other mutants were generated by site-directed mutagenesis, using the Phusion site-directed mutagenesis kit (Thermo Fisher Scientific Inc., Waltham, MA, USA). To co-express WT and mutant NPM-ALK kinase in COS7 or HEK-293T cells, a double cassette vector pBudCE4.1 (Invitrogen) was used, in which WT NPM-ALK was subcloned into the EF-1α multiple cloning site (MCS) and fused to the V5 epitope, whereas NPM-ALK mutants were subcloned into the CMV MCS and fused to *myc* tag.

The full-length human ALK cDNA was purchased from ATCC and subcloned into the mammalian expression vector pcDNA3.1. Point mutations F1174L and R1275Q were introduced by site-directed mutagenesis, as previously indicated.

### Transfection, treatments and immunofluorescence

To evaluate the effects of NPM-ALK mutations generated, exponentially growing HEK-239T cells were transiently transfected with WT and/or mutated NPM-ALK constructs using Lipofectamine 2000 reagent (Invitrogen), according to manufacturer’s instructions. NPM-ALK expression and activity were analyzed in the presence or absence of the ALK-specific inhibitors crizotinib and TAE684, as described in the manuscript.

In the same manner, HEK-293T were transfected with WT or mutated ALK constructs and full-length receptor expression and activity were measured.

To assess localization of WT and mutant NPM-ALK proteins, COS7 cells (0.2 x 10^5^) were plated on 8-well chamber slides, transfected with 0.5 μg of respective plasmids and processed for immunofluorescence as described previously [32].

### Cell lysis, immunoblotting and immunoprecipitation

To assess protein expression and activity, the cells were washed twice in ice-cold 1X phosphate-buffered saline (PBS) and lysed by addition of Triton X-100 sample buffer as reported previously [33]. Binding of WT NPM-ALK to INDEL mutants was performed by incubating protein lysates with 1–2 μg of specific antibodies (α-V5, α-*myc* or α-phospho-ALK) at 4°C overnight, and resulting immunocomplexes to 20 μl of Protein G-Sepharose beads for 2 h at 4°C. The immunoadsorbed pellets were washed 4 times with 1% Triton X-100 lysis buffer and heated at 95°C in 1X reducing Laemmli loading buffer. Aliquots of cell lysates (50 μg) and immunoprecipitates were fractionated by 10% SDS-PAGE and transferred to nitrocellulose membranes for Western blot analysis. Proteins were visualized by chemiluminescence. Films were scanned and analyzed by using image analysis software ImageJ (National Institute of Health, Bethesda, MD, USA).

## Results

### NPM-ALK mutational analysis

*ALK* kinase domain mutational status was investigated in ALCL tumour specimens by 454 amplicon ultra-deep sequencing. About 99,000 sequences aligned with *ALK* exons 22–25 were obtained (mean of 2,234 ± 734 sequences per sample) and an overall of 686 sequence variants, 300 SNPs and 386 INDELs, were detected by Roche AVA software. Among these, a total of 7 SNPs were represented with the same frequency of at least 0.5% on both forward and reverse reads and, thus, accepted, whereas 10 deletions and 3 insertions (INDELs), with the highest detection frequency, were identified and validated by an additional blast-based pipeline specifically designed for INDELs detection and quantification. Of the 7 SNPs detected, 5 were silent point mutations, while 2, namely R335Q (c.1004G>A) and R291Q (c.872G>A), were missense mutations (Table 1, S1 Fig.). With respect to INDELs, 9 variants represented alternative spliced transcripts. Two of them, a deletion of *ALK* exon 25 first 2 nucleotides (c.923-924del) and of the whole exon 23 (c.696-825del), were common to all patients (frequency ranges 0.03–4% and 0.1–12.8% respectively), whereas 7 were expressed in a variable number of patients at lower frequency (~0.5%) (Table 1, S1 Fig.). Finally, in 4/9 INDELs the mutation resulted in an out of frame (OOF) transcript.

**Table 1:** NPM-ALK gene mutations found in ALCL patients by ultra-deep sequencing of exons 22–25.

	cDNA variant	Description	Protein change	Amino Acids	Patients	Frequency
1	c.872G>A	missense point mutation	p.R291Q	680	1/36	0.6%
2	c.1004G>A	missense point mutation	p.R335Q	680	1/36	0.5%
3	c.747C>T	silent point mutation	-	680	1/36	0.8%
4	c.825G>A	silent point mutation	-	680	1/36	0.5%
5	c.894G>A	silent point mutation	-	680	1/36	0.8%
6	c.909C>T	silent point mutation	-	680	1/36	0.5%
7	c.975G>A	silent point mutation	-	680	1/36	0.6%
8	c.923-924del	exon 25 first 2 bases as 3’ splice site	p.D309H-OOF	342	36/36	0.03–4%
9	c.696-825del	exon 23 skipping	p.S232R-OOF	273	36/36	0.1–12.8%
10	c.696-923del	exon 23–24 skipping	p.Δ232–307	604	16/36	0.04–0.6%
11	c.826-923del	exon 24 skipping	p.P276R-OOF	310	18/36	< 0.5%
12	c.733-825del	exon 23 partial deletion, alternative 5’ splice site	p.Δ245–275	649	7/36	< 0.5%
13	c.826-894del	exon 24 partial deletion, alternative 3’ splice site	p.Δ276–299	657	4/36	< 0.5%
14	c.924ins24	intron 24 partial retention, alternative 3’ splice site	p.R308Ins8	688	10/36	< 0.5%
15	c.924ins36	intron 24 partial retention, alternative 3’ splice site	p.R308Ins12	692	4/36	< 0.5%
16	c.924ins106	intron 24 partial retention, alternative 3’ splice site	p.D309H-OOF	312	10/36	0.04–0.6%

Sequence numbering is based on GenBank accession U04946.1.

### R291Q and R335Q point mutations affect NPM-ALK activity and drug sensitivity

**Fig 1:**
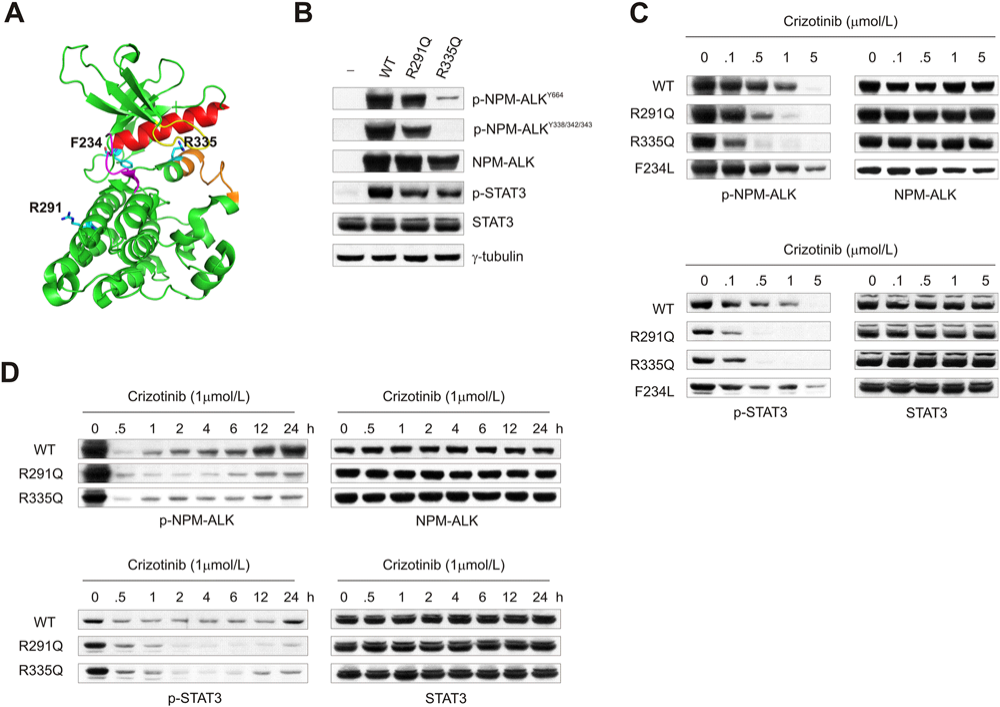
NPM-ALK point mutations R291Q and R335Q affect kinase activity and sensitivity to crizotinib. (**A**) Cartoon representation of ALK kinase domain, showing positions of NPM-ALK R291 (ALK R1231), R335 (ALK R1275) and F234 (ALK F1174) amino acids (PDB 3LCT). Glycin-rich loop, yellow; activation loop, orange; α-C helix, red; hinge region, magenta. (**B**) Relative protein expression and phosphorylation of wild-type (WT) and mutant (R291Q; R335Q) NPM-ALK in HEK-293T transfected cells. The effects of R291Q and R335Q point mutations were assessed on NPM-ALK and STAT3 phosphorylation (p-NPM-ALK^Y664^ or p-NPM-ALK^Y338-342-343^ and p-STAT3, respectively). γ-tubulin was included as loading control. (**C**) Dose-dependent effect of crizotinib on NPM-ALK expression and activity in HEK-293T cells transfected with WT or mutant (R291Q, R335Q, F234L) NPM-ALK constructs. NPM-ALK and STAT3 phosphorylation levels were determined and compared to total protein expression. (**D**) Time-course analysis of NPM-ALK and STAT3 expression and phosphorylation in HEK-293T cells exposed to 1 μM crizotinib over 24 h.

Deep sequencing of NPM-ALK kinase domain identified 2 missense mutations in 2 distinct samples, representing the amino acid changes R335Q and R291Q. Residue R335 lied within the activation loop of ALK kinase domain and corresponded to amino acid R1275 in full-length ALK receptor, whereas R291, corresponding to ALK R1231, was localized on the C-terminal lobe (Fig. 1A). To find out more about these 2 point mutations, we generated NPM-ALK R291Q and R335Q constructs and transfected HEK-293T cells. We found that NPM-ALK R291Q displayed a tyrosine phosphorylation level similar to its WT counterpart, whereas the R335Q mutation markedly reduced ALK kinase phosphorylation both at C-terminal (Y664) and in the activation loop (Y338/342/343) (Fig. 1B). In contrast, downstream STAT3 target phosphorylation was reduced at similar extent, suggesting that both mutants had a lower signalling potential compared to WT NPM-ALK kinase. Indeed, when exposed to crizotinib, a more pronounced dose- and time-dependent inhibitory effect was observed with respect to WT NPM-ALK, which in turn exhibited a progressive recovery overtime (Fig. 1C and D, p-NPM-ALK and p-STAT3). Conversely, the introduction of F234L mutation markedly reduced NPM-ALK drug sensitivity (Fig. 1C), in line with the activating nature of this mutation [12]. Exposure of cells to a second ALK inhibitor, NVP-TAE684 (TAE684), confirmed these observations (S2 Fig.), providing additional evidence of the different enzymatic properties imparted by these mutations to NPM-ALK kinase activity.

### R1275Q-induced structural changes affect ALK receptor activity

**Fig 2:**
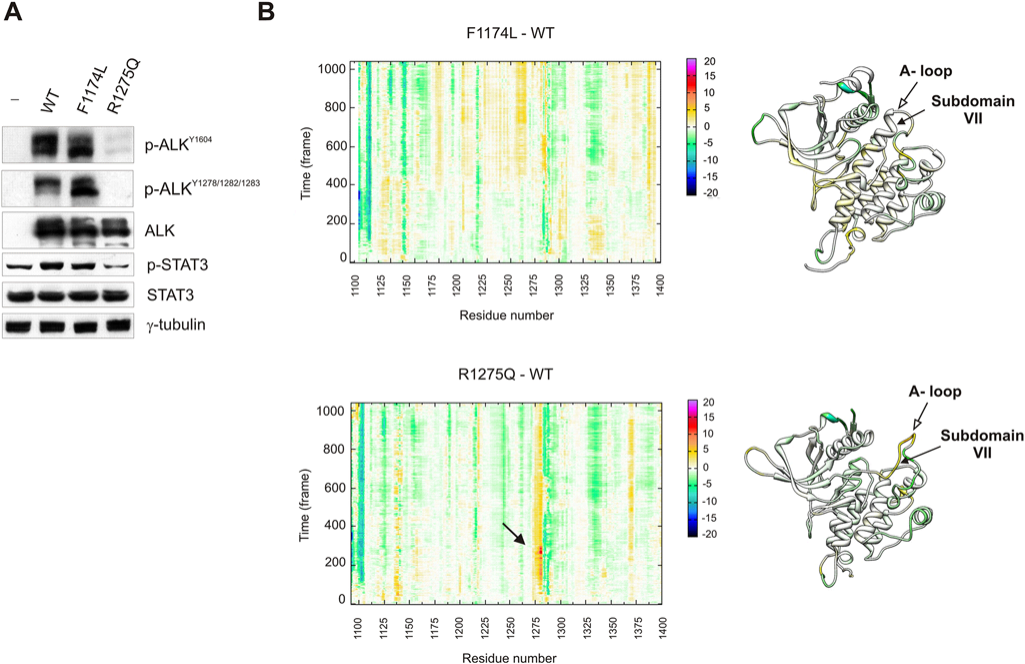
F1174L and R1275Q molecular dynamics simulation. (**A**) Effect of F1174L (NPM-ALK F234L) and R1275Q (R335Q) point mutations on full-length ALK receptor expression (ALK), phosphorylation (p-ALK^Y1604^ and ^Y1278-1282-1283^) and signalling (p-STAT3) in HEK-293T cells. (**B**) Alpha carbon "Rainbow Differential RMSD analysis" between F1174L or R1275Q ALK KD and WT over time (1000 ns) (RMSD_F1174L—RMSD_WT, *upper panel*; RMSD_R1275Q—RMSD_WT, *lower panel*). The differential RMSD of each position is encoded by a chromatic scale: negative values (from green to blu) identify regions in which the WT ALK KD is more flexible than the mutant one; positive values (from yellow to magenta) identify regions in which the WT KD is less flexible than mutant KD. Cartoon representation of ALK KD colored by the corresponding differential RMSD values (RMSD_mutant—RMSD_WT) are shown on the right.

In the wide spectrum of ALK mutations, R1275Q and F1174L are the most frequently reported mutations in cancer that result in dysregulation of ALK activity and signalling [9,34]. However, while F1174L is a point mutation that enhances ALK kinase activity and oncogenic potential *per sé* [12], gain-of-function properties of R1275Q are less clear and somehow depend on the model system employed [9,35]. Therefore, to confirm our findings, we introduced F1174L and R1275Q mutations into full-length ALK background and expressed the corresponding constructs into HEK-293T cells. As for F234L and R335Q mutations in NPM-ALK, F1174L ALK exhibited high levels of phosphorylation when ectopically expressed, whereas R1275Q ALK was poorly phosphorylated and less active (p-STAT3) compared to WT and F1174L kinases (Fig. 2A). Besides, ligand-independent receptor activation resulting from protein overexpression, as observed for WT ALK, was not noted in R1275Q ALK expressing cells, providing further evidences of the kinase defective nature of this mutant [36]. Indeed, molecular dynamics simulation analysis of F1174L and R1275Q ALK kinase domain demonstrated significant perturbations in the kinase domain of R1275Q ALK compared to WT (Fig. 2B, R1275Q—WT, closed arrowhead) coupled to a high grade of misfolding of the activation loop moiety (Fig. 2B, cartoon aside, A-loop and Subdomain VII α-helix, open and close arrowheads, respectively; S1 Movie and S2 Movie: F1174L in yellow, R1275Q in red, WT in grey).

### Molecular modelling and expression analysis of NPM-ALK INDEL variants

By using an approach of cDNA-based amplicon sequencing, we could detect the presence of NPM-ALK alternative spliced transcripts as well. As mentioned above, we identified 13 INDEL mutations, 9 of which resulting from either exon skipping or partial intron retention (Table 1). In 6 of 9 splicing variants, the mutations were associated with extensive deletion of regions crucial for ALK kinase activity, while in 3 variants the structural rearrangements were compatible with ALK enzymatic activity. We focused on the 2 most common deletions found in patients (c.923-924del, p.D309H-OOF; c.696-825del, p.S232R-OOF) and on the 3 in frame INDELs that conserved most of the residues critical for ATP binding and hydrolysis (c.826-894del, p.Δ276–299; c.924ins24, p.R308-Ins8; c.924ins36, p.R308-Ins12) (S3 Fig.).

**Fig 3:**
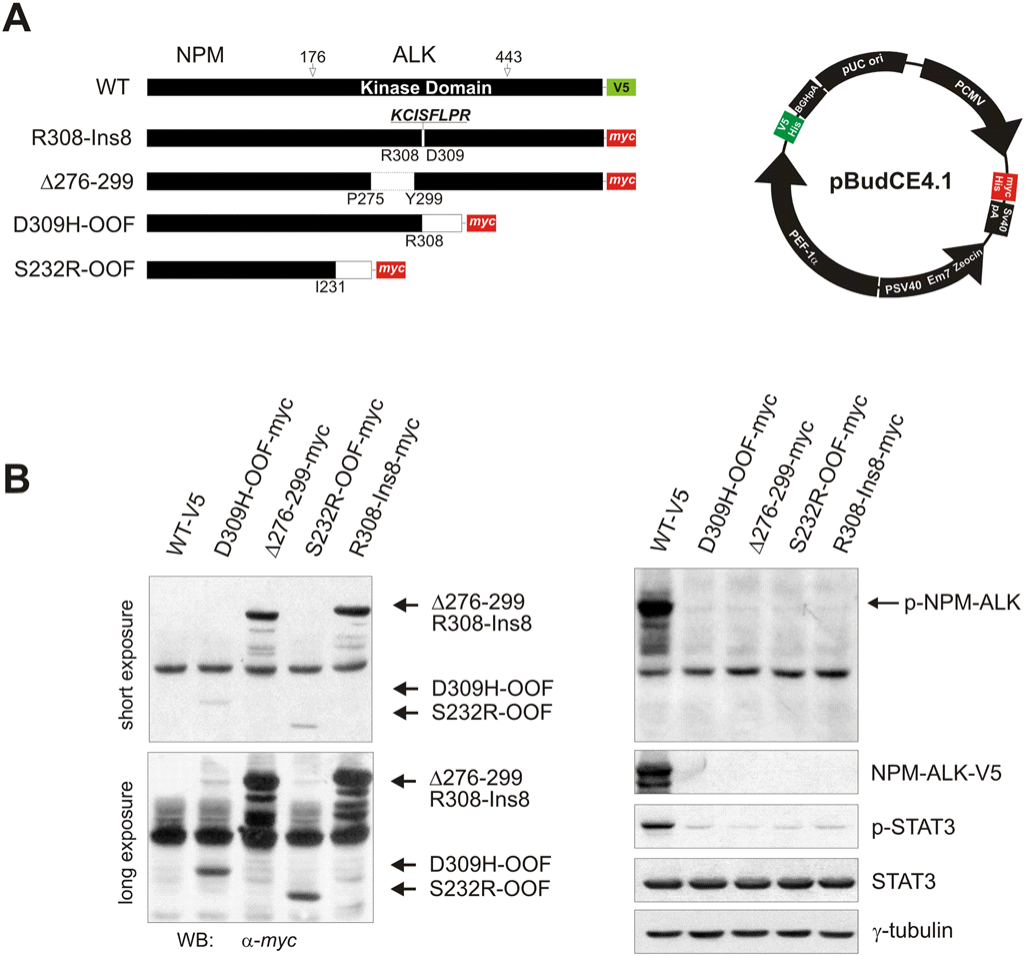
NPM-ALK INDEL mutants expression and activity in HEK-293T cells. (**A**) Schematic representation of NPM-ALK constructs generated into pBudCE4.1 expression vector according to the *in vivo* mutational analysis. The figure represents each NPM-ALK construct with the corresponding INDEL (*left*) and summarizes the features of the pBudCE4.1 vector (*right*). (**B**) Protein expression analysis of WT and D309H-OOF, Δ276–299, S232R-OOF or R308-Ins8 NPM-ALK constructs in HEK-293T cells by Western blotting. Mutants were detected using an anti-*myc* specific antibody (NPM-ALK-*myc*, *left panels*), whereas WT NPM-ALK was visualized with an anti-V5 antibody (NPM-ALK-V5, *right panels*). NPM-ALK and STAT3 phosphorylation was also measured by immunoblotting (*right panels*, p-NPM-ALK and p-STAT3, respectively), using γ-tubulin as loading control.

To examine the functional implication of these variants, each mutant was subcloned and tagged using a double cassette vector designed for simultaneous expression of 2 genes, and expression was assessed in the presence or absence of recombinant WT-V5 NPM-ALK (Fig. 3A). As shown in figure, all mutants were expressed in HEK-293T cells, although low expression levels were observed for extensively deleted S232R-OOF and D309H-OOF INDELs (Fig. 3B, *left panels*, short and long exposure). Besides, all mutants were catalytically inactive (Fig. 3B, *right panels*, p-NPM-ALK and p-STAT3), including those initially predicted to be functional based on their conserved sequences. These results were unexpected particularly for R308-Ins8 and R308-Ins12, since the insertions preserved all determinants crucial for ALK kinase activity, including the catalytic Asp308 and the adjacent Arg307 residue (S4A Fig., *R* and *D* in colour). Therefore, to provide a molecular explanation to these findings, we performed molecular dynamics simulation on R308-Ins8 and R308-Ins12 mutants and compared time-dependent conformational changes of mutant KD to that of WT NPM-ALK. While WT kinase showed major conformational changes in the P-loop moiety during the selected time frame (S4B Fig.), R308-Ins8/12 exhibited a pronounced modification of the A-loop in close proximity to the inserted regions (S4C–D Fig., *right panels*). These structural rearrangements supported novel polar interactions between Ins8/12 motifs and key residues for ATP binding and hydrolysis, having an inhibitory influence on the whole catalytic process (S4C–D Fig., *left panels*).

### *myc*-tagged INDELs bind and inactivate V5-tagged NPM-ALK kinase

In cells co-expressing native and catalytically inactive alleles, the formation of heterocomplexes may impact on native protein activity. A kinase inactive mutant, in fact, exhibits a dominant-negative effect on the active allele but also can interfere with drug-induced kinase inhibition [37].

**Fig 4:**
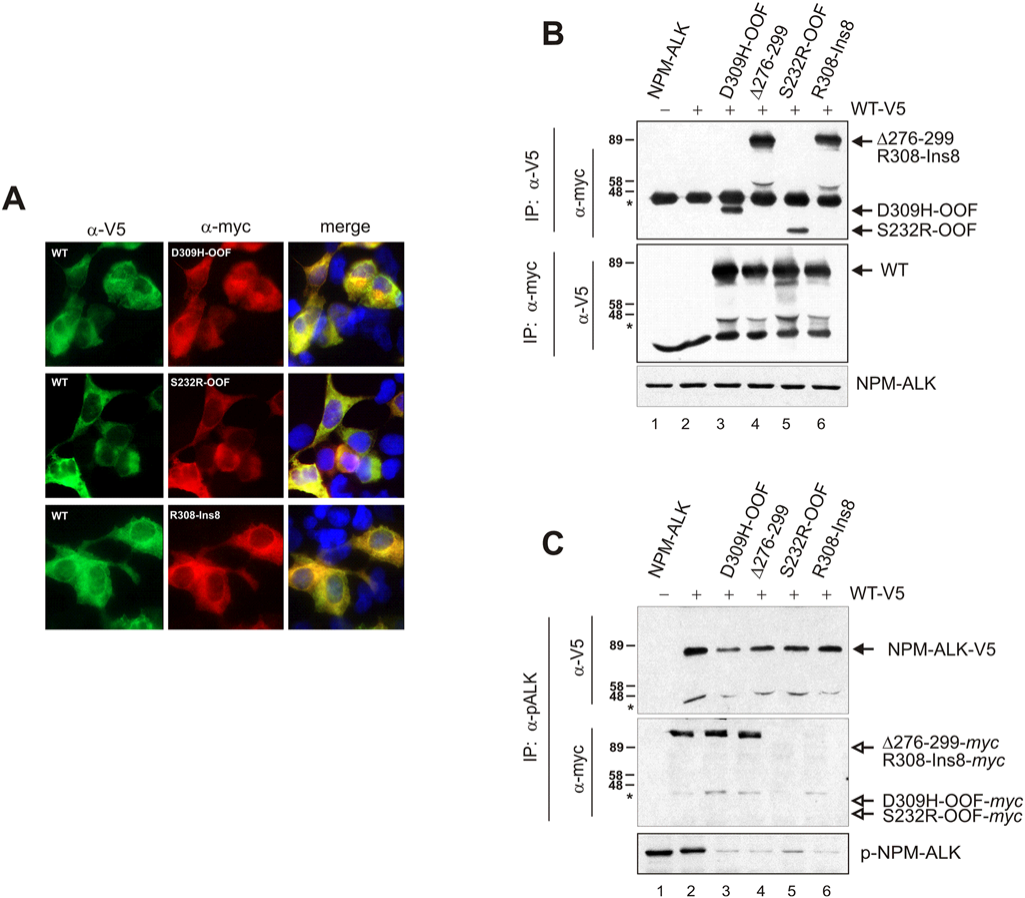
INDEL mutants exert a dominant negative effect on wild-type NPM-ALK kinase activity. (**A**) Subcellular co-localization (*merge*) of WT NPM-ALK (α-V5, green) and D309H-OOF, S232R-OOF or R308-Ins8 mutants (α-*myc*, red) in COS7 cells. Cell nuclei are in blue (DAPI dye). (**B**) NPM-ALK WT/INDEL complex formation in HEK-293T co-transfected cells (+), by reciprocal immunoprecipitation. Anti-V5 and anti-*myc* antibodies were used to precipitate WT NPM-ALK (*upper panel*) or D309H-OOF, Δ276–299, S232R-OOF and R308-Ins8 mutants (*middle panel*), respectively, before reciprocal immunoblotting. Untagged WT NPM-ALK (*lane 1*) was also expressed in these cells and used for quality control of non-specific binding. Total NPM-ALK expression is shown in the lower panel. (**C**) Immunoprecipitation of active NPM-ALK with anti-p-ALK^Y1604^ antibody (α-pALK) in co-transfected (+) HEK-293T cells. Phospho-ALK immunocomplexes were probed for V5- (α-V5, WT NPM-ALK) or *myc*-tagged (α-*myc*, INDELs) proteins. Compared to WT NPM-ALK (*upper panel*, closed arrowhead), INDEL mutants are not phosphorylated (*middle panel*, lanes 3–6, open arrowheads), thought they reduce basal phosphorylation of WT NPM-ALK kinase (*upper* and *lower panels*). Asterisks indicate immunoglobulin (IgG) heavy chain, whereas arrows distinguish relative protein position.

To functionally test this hypothesis, we introduced WT NPM-ALK and INDEL mutants into HEK-293T cells (Fig. 4A) and assessed NPM-ALK kinase activity upon simultaneous expression. Fresh cell lysates were then immunoprecipitated using either anti-V5 or-*myc* antibodies, and reciprocal immunoblottings were performed. As expected, *myc*-tagged mutants associated with V5-tagged NPM-ALK (Fig. 4B, *upper panel*), and *vice versa* (Fig. 4B, *middle panel*), irrespective of their mutational status and activity. However, when a phospho-specific ALK antibody was used to purify expressed proteins, the immunocomplexes contained WT-V5 NPM-ALK (Fig. 4C, *upper panel*, closed arrowhead) but not *myc*-tagged proteins (Fig. 4C, *middle panel*, open arrowheads). Indeed, higher levels of phospho-NPM-ALK were found in cells expressing WT NPM-ALK kinase alone (Fig. 4C, *lower panel*). These data demonstrated that INDELs exert a dominant-negative effect on WT NPM-ALK kinase throughout the formation of inactive protein heterocomplexes.

### NPM-ALK INDELs expression increases native NPM-ALK sensitivity to crizotinib

Finally, to investigate whether INDELs may affect drug sensitivity, HEK-293T cells expressing WT NPM-ALK, either alone or in combination with *myc*-tagged mutants, were exposed to increasing concentrations of crizotinib, and steady-state of total and phosphorylated NPM-ALK was assessed.

**Fig 5:**
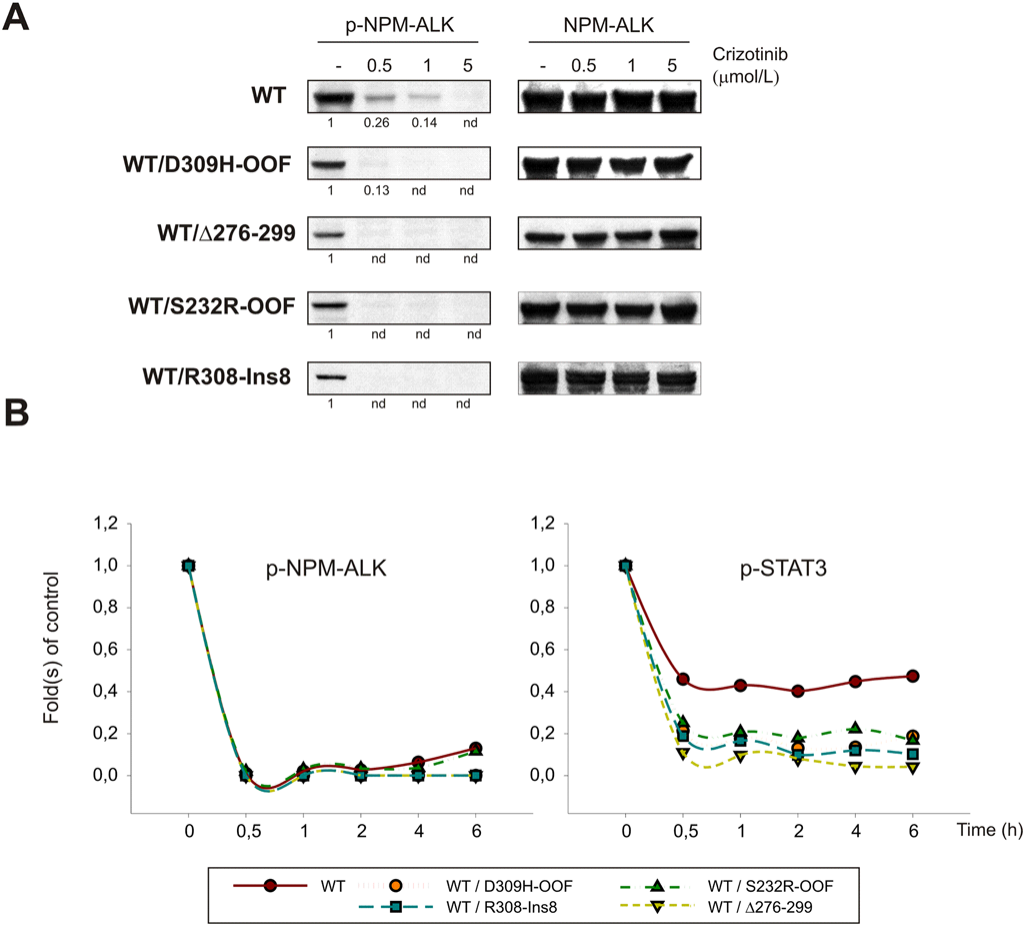
INDEL mutants increase NPM-ALK sensitivity to crizotinib. HEK-293T cells were transfected with WT NPM-ALK alone or in combination with D309H-OOF, Δ276–299, S232R-OOF and R308-Ins8 mutants. (**A**) Total and phosphorylated NPM-ALK levels are shown before and after exposure to crizotinib (0.5, 1 and 5 μM) for 6 hours, and band densities, where indicated, are reported as folds of control (-). (**B**) Crizotinib inhibits NPM-ALK phosphorylation and downstream signalling in a time-dependent manner. NPM-ALK was expressed in HEK-293T cells either alone (WT) or in combination with INDEL mutants (WT/D309H-OOF, WT/Δ276–299, WT/S232R-OOF, WT/R308-Ins8) and exposed to 1 μM Crizotinib for increasing time intervals. Steady-state of phosphorylated NPM-ALK (p-NPM-ALK) and STAT3 (p-STAT3) proteins was determined by Western blotting and reported in graph as fold(s) of control (time exposure 0).

Immunoblotting experiments showed a significantly higher sensitivity of WT NPM-ALK to crizotinib in cells co-transfected with mutated NPM-ALK constructs, as co-expression led to a complete inhibition of NPM-ALK phosphorylation (p-NPM-ALK) even at the lowest dose administered (Fig. 5A). Consistently, time-dependent NPM-ALK inhibition showed a brisk reduction of kinase autophosphorylation after 30’ of drug exposure, both in the presence or absence of inactive INDELs (Fig. 5B, *left*). However, a progressive recovery of phosphorylated NPM-ALK was observed in HEK-293T cells expressing either WT alone or in combination with S232R-OOF mutant, providing additional evidence of a stronger NPM-ALK activity when expressed alone or with unstable mutants. In accordance with these observations, drug-induced inhibition of NPM-ALK-dependent STAT3 phosphorylation was more prominent when INDEL mutants were co-expressed, due to the higher amount of non-functional heterodimers formed (Fig. 5B, *right*).

## Discussion

Tyrosine kinase inhibitors have become the gold standard therapy of tumour types expressing oncogenic forms of protein tyrosine kinases. However, clinical studies indicate that a significant portion of patients treated with tyrosine kinase inhibitors develop clinical resistance, due to the selection of cancer cells carrying mutations on target kinase.

In NSCLC, primary resistance mechanism of ALK-fusion positive tumours has been mostly secondary mutations within the kinase domain of EML4-ALK, either when compromising drug binding (L1196M, G1269A) or when affecting enzyme conformation and activity (C1156Y, I1171T) [11,38]. In IMT tumours, instead, acquired resistance to ALK inhibitors has shown to occur upon clonal selection of cells harbouring the F1174L activating mutation, in virtue of the important effects this substitution has on ALK tertiary structure [12]. NSCLC and IMT tumours harbouring these 2 types of mutation, however, do not display significant changes in ALK constitutive activity and, therefore, might be positively selected *in vivo* only in the presence of ALK inhibitors. In contrast, missense mutations associated with familial and sporadic neuroblastoma are mainly activating mutations affecting ALK kinase activity and transforming capability and their detection can occur at diagnosis [39]. The prognostic significance of these mutations, however, is not known yet [13], although for some (G1128A, I1171N, F1174L and F1174V) compound sensitivity has been predicted or experimentally tested, or reported in other tumour types (i.e. F1174V in NSCLC and F1174L in IMT) [12,38]. These findings suggest that acquired resistance mutations likely occur in oncogene-driven malignancies, whereas activating site substitutions are more frequent in oncogene-positive tumours. However, extensive clinical experience with BCR-ABL inhibitor imatinib has proven that at least a portion of relapsing patients already harbour the same relapse mutations at diagnosis [20,40,41,42].

In the last few years, the advent of massive parallel next-generation sequencing technologies has greatly enhanced the scope and the speed of molecular cancer research, offering a powerful solution for mutation discovery in tumour samples when mutations are not “fixed” and resistant clones have not emerged yet [21,43]. Early detection of resistance mutations is, therefore, important to predict response to treatment, but also for the impact their identity, frequency and evolution have on clinical course.

Taking advantage of ultra-deep sequencing technology, we amplified exons 22–25 from *ALK* cDNA, to ensure high sensitive detection of mutations from the expressed translocated *NPM-ALK* allele in ALCL patients. We aimed at discovering pre-existing NPM-ALK mutations, when also barely expressed, to evaluate their potential impact on NPM-ALK transforming activity and drug inhibitor sensitivity.

Two point mutations were identified at diagnosis in 2 distinct cases: a G→A transition at nucleotide 872 and a G→A substitution at nucleotide 1004, which corresponded to R291Q and R335Q amino acid substitution, respectively. R291Q and R335Q corresponded to full-length ALK R1231Q and R1275Q substitutions in neuroblastoma patients [39,44]. We showed here that both residue mutations affected NPM-ALK signalling and drug inhibitor sensitivity. R291Q NPM-ALK kinase displayed catalytic activity similar to WT kinase, whereas R335Q mutation was shown to decrease NPM-ALK autophosphorylation capacity. These data corroborated previous published observations [26,34,35], although they were different from the putative activating nature of this substitution assigned by others [9,44,45]. However, when R1275Q residue mutation was generated in full-length ALK receptor kinase, catalytic activity was reduced compared to WT and F1174L, due to conformational changes in the active site cavity, as sustained by molecular dynamics simulation.

Although our principal aim was the identification of KD mutations potentially relevant for acquired drug resistance, our approach of cDNA-based ultra-deep sequencing provided information on alternative-spicing events in NPM-ALK kinase domain as well. Herein, we identified INDEL mutations resulting from exons skipping or partial intron retention events, including common deletions and more rare in-frame variants previously described in other tumour types [46,47]. An important question addressed in this study was how INDELs expression could affect NPM-ALK activity and drug responsiveness, since oncogenic function of NPM-ALK is strictly dependent on its own dimerization and trans-phosphorylation capacity. Previous studies have shown that insertion/deletion aberrations may have profound effects on protein function, affecting kinase activity or drug-binding affinity. In NSCLC tumours, recurrent EGFR mutations are localized within the catalytic domain and comprise both INDELs and point mutations. However, whereas point mutations are usually activating, INDELs may favor or not the active state of EGFR kinase, depending on size and position. Indeed, whereas NSCLC patients with exon 19 insertions or deletions are responsive to EGFR inhibitors, in-frame insertions in exon 20 confer drug resistance both *in vitro* and *in vivo* [48,49]. Both types of mutation are likely to stabilize the active conformation of the kinase. However, while exon 19 insertions increase EGFR affinity for its inhibitors, insertions in exon 20 result in a significant reduction of it [50]. As a consequence, patients with INDELs resulting in reduced EGFR kinase activity are among the best responders to EGFR inhibitors and have a favourable outcome during treatment [51,52].

In this scenario, we found that all INDELs had a dominant-negative effect on WT NPM-ALK and, by forming nonfunctional heterodimers, they significantly reduced the overall kinase activity while increasing sensitivity to specific inhibition. In cells expressing WT NPM-ALK, signal processing was strongly reduced, albeit minimally maintained. In cells co-expressing NPM-ALK kinase and INDEL variants, instead, cell signalling was totally averted both in a time- and dose-dependent manner.

In summary, our study demonstrated that NPM-ALK mutations are uncommon in ALCL patients at diagnosis. These mutations result in single amino acid substitutions or more complex structural rearrangements of NPM-ALK kinase. Whether these subclonal mutations coexist in the same cell with the WT allele, expand or are lost for natural selection is not know yet. However, their identification and characterization may be helpful to identify the most appropriate therapy for each patient, preventing either over-treatment or relapse.

## Supporting Information

S1 Fig Raw flowgrams from individual reads showing *NPM-ALK* single nucleotide substitution (1004G>A; 872G>A), deletion (923-924del; 826-894del; 696-825del) or insertion (924ins24) variants as detected *in vivo* by 454 analysis. All the corresponding amino acid changes are described in Table 1. Single nucleotide substitutions are in red; deletions are represented by grey shaded regions; inserted nucleotides are in red within grey shaded regions.
(TIF)

S2 Fig Sensitivity of WT or mutant NPM-ALK to NVP-TAE684 (TAE684) small-molecule inhibitor. HEK-293T cells were transfected with WT or mutated (R291Q, R335Q, F234L) NPM-ALK constructs and treated with increasing concentrations of TAE684 for 6h. NPM-ALK and STAT3 phosphorylation levels were assessed by immunoblotting and compared to total protein expression. γ-tubulin was used as loading control.
(TIF)

S3 Fig (**A**) Amino acid sequence alignments of WT NPM-ALK (black) and alternative splicing variants R308-Ins8 (red), R308-Ins12 (orange), Δ276–299 (green), D309H-OOF (blue) and S232R-OOF (purple). Amino acid substitutions caused by frameshift mutations are shown in colour; premature stop codons are indicated by asterisks; Arg308 and Asp309 are underscored (*R* and *D*, respectively). (**B**) Cartoon representation of ALK KD (green) showing positions of deletions and insertions (red) of D309H-OOF, S232R-OOF, Δ276–299 and R308Ins8/12 ALK variants as obtained by MOE homology analysis.
(TIF)

S4 Fig (**A**) Sequence of ALK catalytic loop, with catalytic aspartic acid (*D*) residue and upstream basic arginine (*R*) in red. Below are the corresponding sequences of R308-Ins8 and—Ins12 mutants, in which the Arg-Asp motif is maintained by the arginine residue (green) of the inserted sequence. (**B**) Alpha carbon Rainbow RMSD analysis of ALK kinase domain (WT) over time (200 ns), with relative conformational changes of the P-loop and A-loop indicated. (**C**) R308-Ins8 and (**D**) R308-Ins12 RMSD analysis (*right panels*) with snapshots of the **Ins8** and **Ins12** conformational changes occurring. Left panels show a novel polar interaction occurring between R316 and D338 of the DFG motif in the Ins8 mutant, and the formation of a salt-bridge between residues D220 and K313 (Ins12) and a H-bond between A186 and K313 in the R308-Ins12 kinase domain moiety (P-loop, white; αC-helix, red; Ins8/12, blue; A-loop, yellow).
(TIF)

S1 Movie Molecular dynamics simulation analysis of ALK-F1174L kinase domain (yellow) compared to WT (grey). (MP4)

S2 Movie Molecular dynamics simulation analysis of ALK-R1275Q kinase domain (red) compared to WT (grey). (MP4)

S1 Table Sequences of fusion primer pairs used for ALK exons 22–25 amplification in 454 analysis. Target-specific sequences are represented in bold.
(XLS)

S2 Table Samples analyzed by 454 amplicon sequencing, with corresponding Multiplex Identifiers (MID). (XLS)
