# Supplementary figures and images for: ALK Kinase Domain Mutations in Primary Anaplastic Large Cell Lymphoma: Consequences on NPM-ALK Activity and Sensitivity to Tyrosine Kinase Inhibitors
Source: PLoS One. 2015 Apr 13;10(4):e0121378. doi: 10.1371/journal.pone.0121378 (PMC4395299; doi:10.1371/journal.pone.0121378)

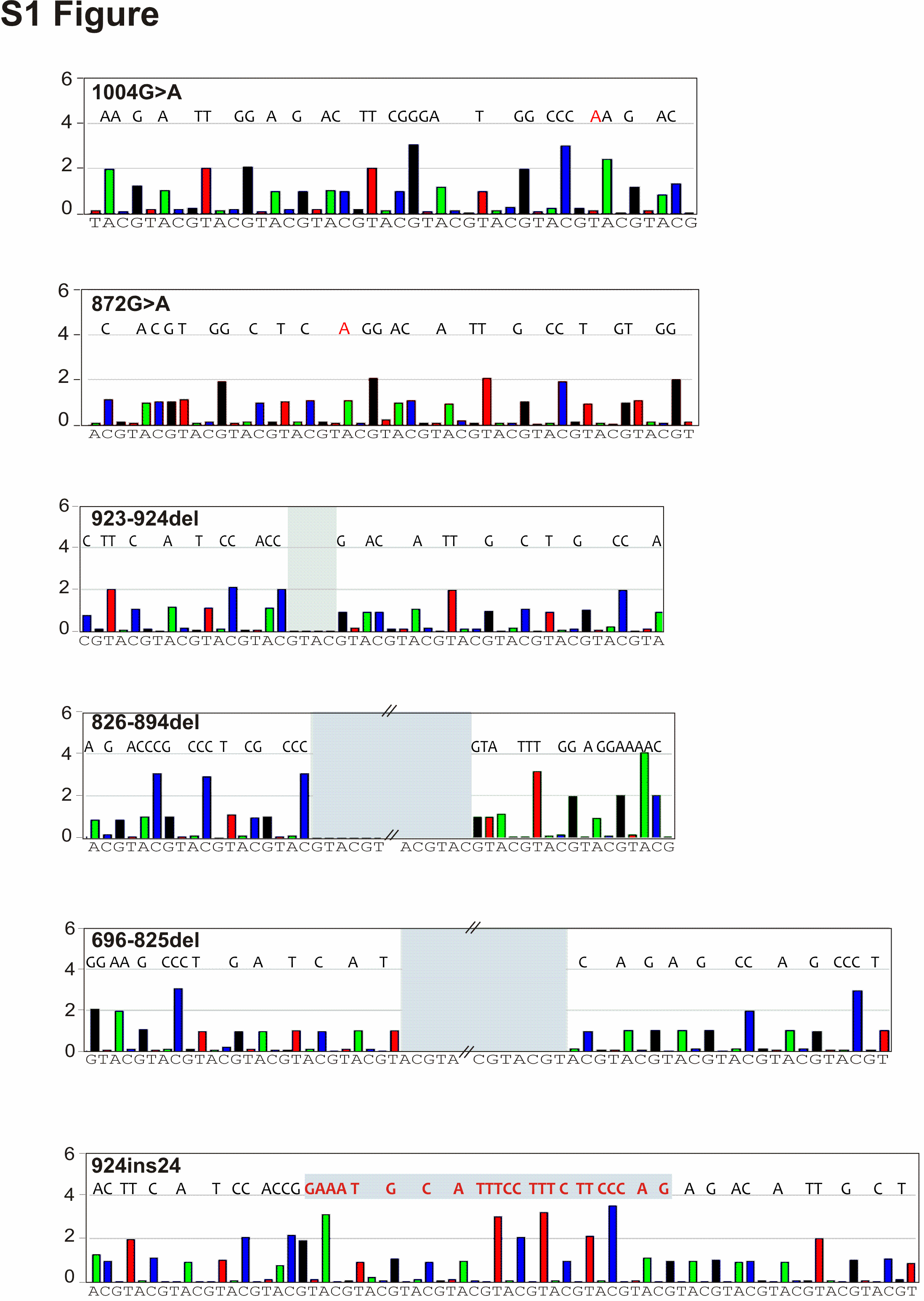

Supplement: S1 Fig — All the corresponding amino acid changes are described in Table 1. Single nucleotide substitutions are in red; deletions are represented by grey shaded regions; inserted nucleotides are in red within grey shaded regions. (TIF) [file pone.0121378.s001.tif]

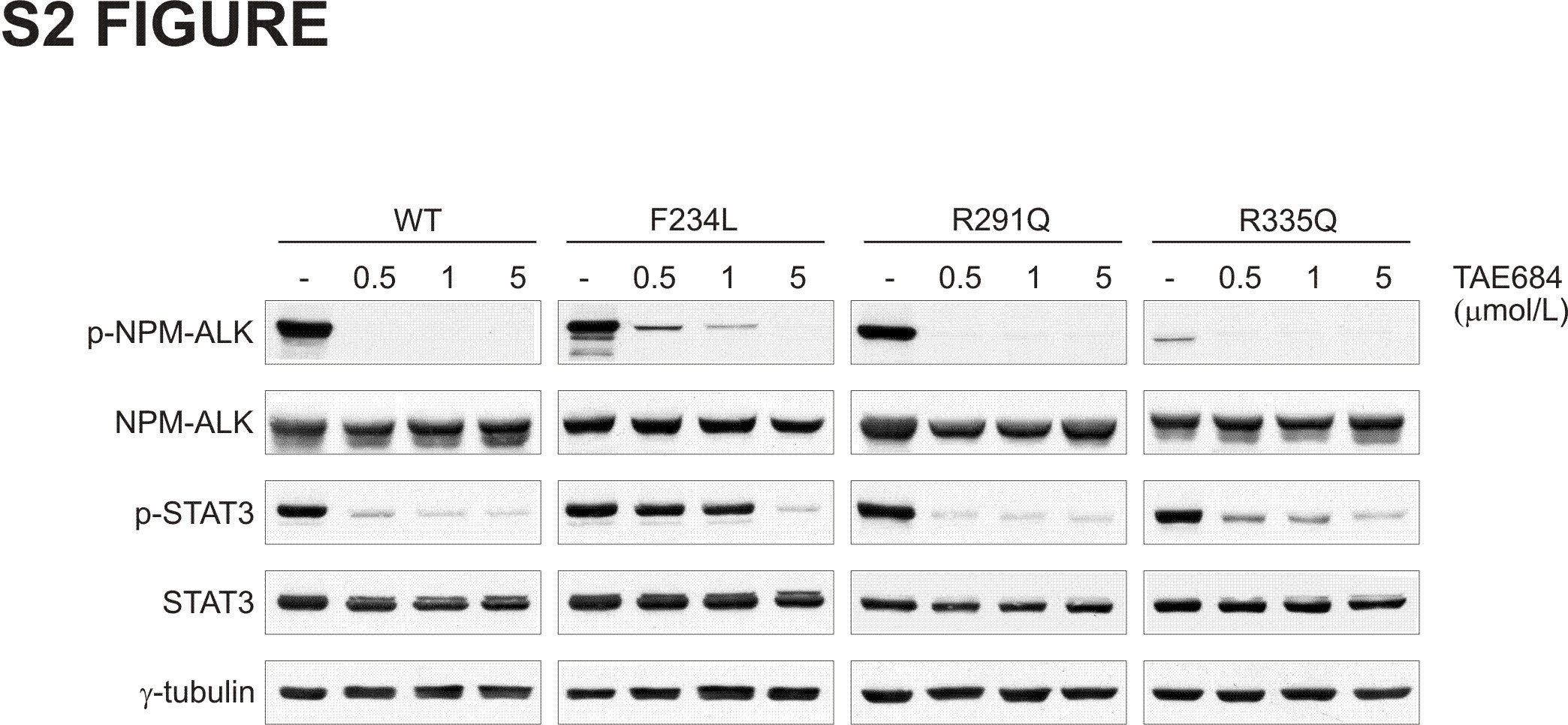

Supplement: S2 Fig — NPM-ALK and STAT3 phosphorylation levels were assessed by immunoblotting and compared to total protein expression. γ-tubulin was used as loading control. (TIF) [file pone.0121378.s002.tif]

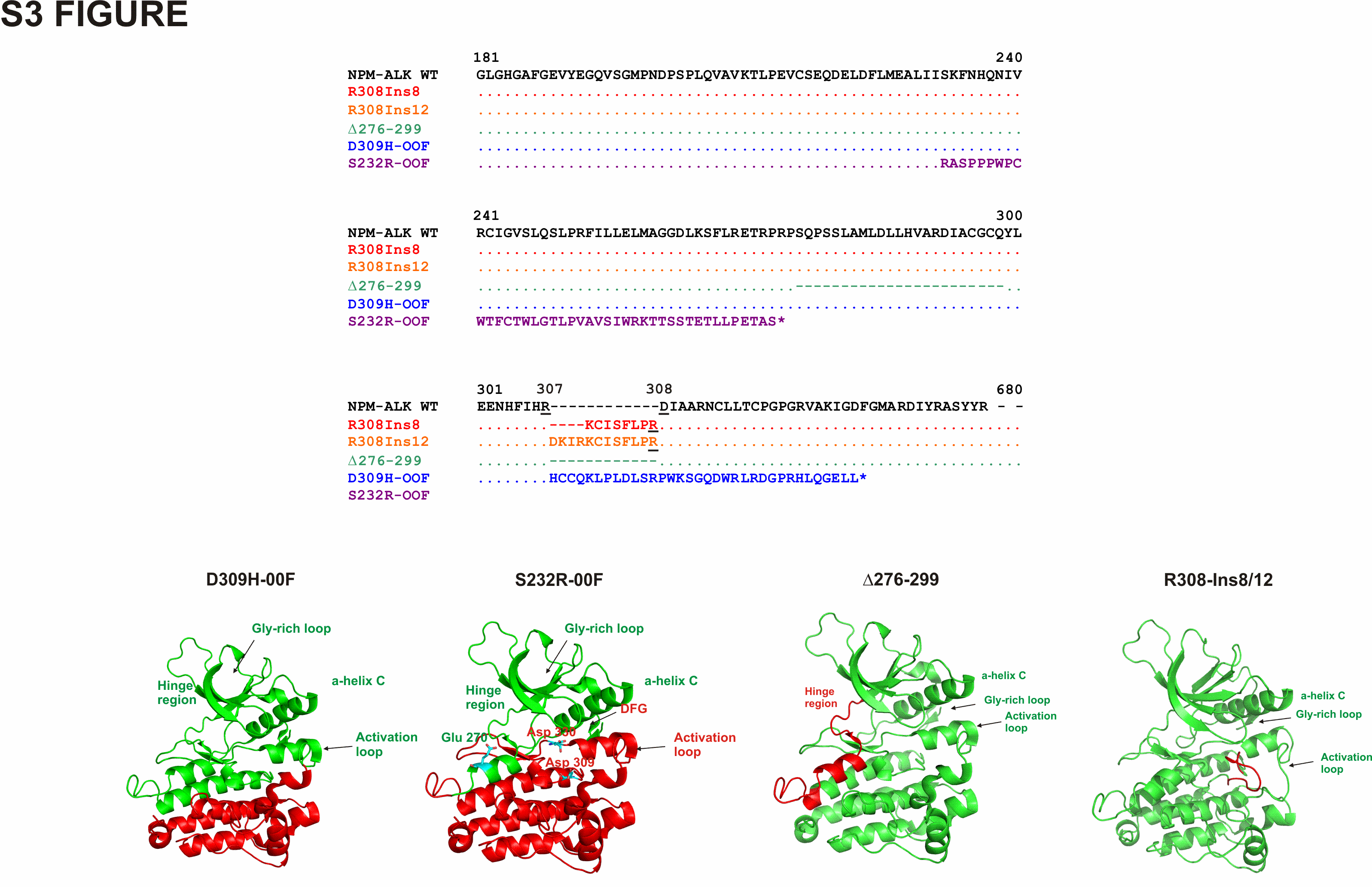

Supplement: S3 Fig — (A) Amino acid sequence alignments of WT NPM-ALK (black) and alternative splicing variants R308-Ins8 (red), R308-Ins12 (orange), Δ276–299 (green), D309H-OOF (blue) and S232R-OOF (purple). Amino acid substitutions caused by frameshift mutations are shown in colour; premature stop codons are indicated by asterisks; Arg308 and Asp309 are underscored (R and D, respectively). (B) Cartoon representation of ALK KD (green) showing positions of deletions and insertions (red) of D309H-OOF, S232R-OOF, Δ276–299 and R308Ins8/12 ALK variants as obtained by MOE homology analysis. (TIF) [file pone.0121378.s003.tif]

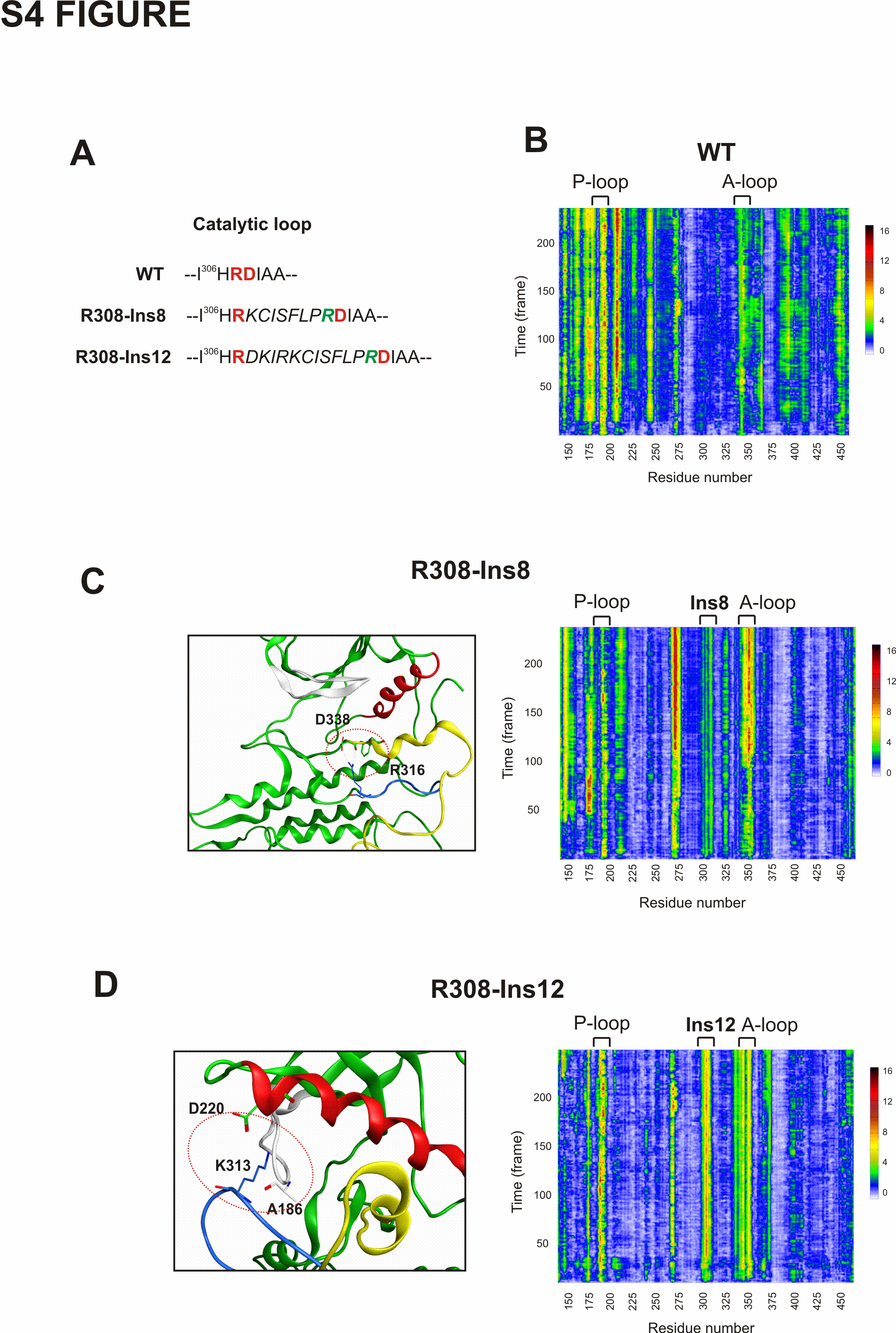

Supplement: S4 Fig — (A) Sequence of ALK catalytic loop, with catalytic aspartic acid (D) residue and upstream basic arginine (R) in red. Below are the corresponding sequences of R308-Ins8 and—Ins12 mutants, in which the Arg-Asp motif is maintained by the arginine residue (green) of the inserted sequence. (B) Alpha carbon Rainbow RMSD analysis of ALK kinase domain (WT) over time (200 ns), with relative conformational changes of the P-loop and A-loop indicated. (C) R308-Ins8 and (D) R308-Ins12 RMSD analysis (right panels) with snapshots of the Ins8 and Ins12 conformational changes occurring. Left panels show a novel polar interaction occurring between R316 and D338 of the DFG motif in the Ins8 mutant, and the formation of a salt-bridge between residues D220 and K313 (Ins12) and a H-bond between A186 and K313 in the R308-Ins12 kinase domain moiety (P-loop, white; αC-helix, red; Ins8/12, blue; A-loop, yellow). (TIF) [file pone.0121378.s004.tif]
